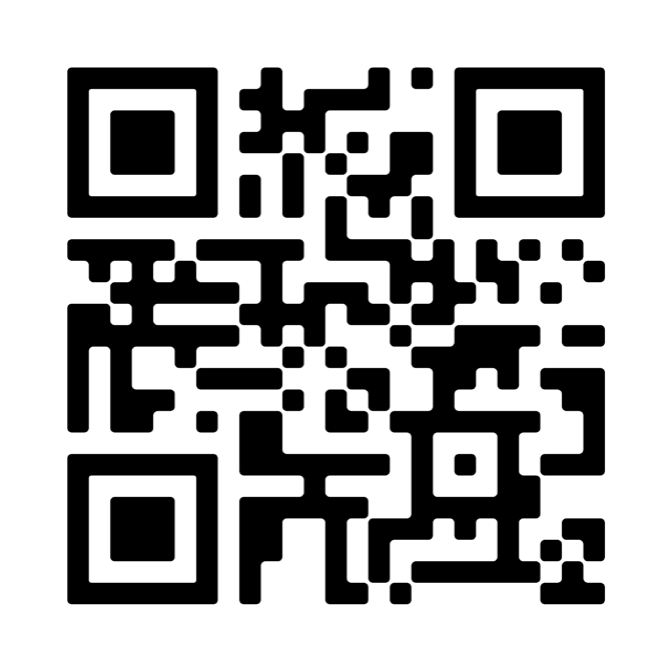# Early approach for the iatrogenic vesico-vaginal fistula repair: a video case report

**DOI:** 10.52054/FVVO.16.2.014

**Published:** 2024-06-28

**Authors:** M Afonina, S Waligora Lages, A Liori, R Botchorishvili

**Affiliations:** San Paolo University Hospital, Department of Obstetrics and Gynaecology, Via Antonio di Rudinì, 8, 20133, Milan, Italy; Department of Gynaecological Surgery, CHU Estaing Clermont-Ferrand, 1 Place Lucie Aubrac, 63000 Clermont-Ferrand, France; Hospital Israelita Albert Einstein, Department of Obstetrics and Gynaecology, Avenida Albert Einstein, 627|701, Morumbi – Sao Paulo, Brazil; General Maternal Hospital of Elena Venizelou, Elena Venizelou 2, Athens 11521, Greece

**Keywords:** Vesico-vaginal fistula, early repair, minimally invasive approach

## Abstract

**Background:**

Vesico-vaginal fistula (VVF) is a rare but debilitating condition, characterised by an abnormal connection between the bladder and vagina. While obstetric-related cases prevail in developing countries, iatrogenic fistulas are more common in industrialised ones, often resulting from pelvic surgeries.

**Objectives:**

The optimal timing for surgical correction of VVF remains debated, often leaning towards delayed intervention. Here we report a successful early laparoscopic repair of an iatrogenic VVF following hysterectomy.

**Materials and Methods:**

The patient, a 54-year-old woman, presented with VVF after a hysterectomy. The laparoscopic repair was performed promptly upon diagnosis.

**Main outcome measures:**

To assess the feasibility and effectiveness of an early repair of a gynaecological-related VVF.

**Results:**

First, cystoscopy identified the bladder edge of the VVF. Second, laparoscopy was performed and the vesico-vaginal dissection was carried out. The excision of the previous stitches and of the fibrotic tissue was undertaken to create free flaps for suturing. The bladder was repaired in a double layer, and a single layer was applied to the vagina. Finally, the omentoplasty was done. The patient was discharged on postoperative day 5. No complications occurred.

**Conclusions:**

This successful case demonstrates the feasibility and safety of early laparoscopic repair for gynaecological surgery-related vesico-vaginal fistulae. While acknowledging the need for further studies to standardise techniques, this report contributes to the evolving understanding of optimal management for this complex condition.

## Learning objective

A vesico-vaginal fistula (VVF) is an abnormal connection between the bladder and vagina. VVF may manifest following obstetric events, pelvic surgery, radiation therapy, or gynaecological malignancies. There is currently no consensus regarding the optimal timing for their surgical correction. In this report, we present a successful early intervention for gynaecological surgery related VVF.

## Introduction

Vesico-vaginal fistula (VVF) is defined as an abnormal connection between the bladder and the vagina and affects over 3 million women worldwide (Núñez Bragayrac et al., 2015). It can be classified according to its aetiology (obstetric- related or iatrogenic) and complexity (simple or complex).

VVFs are generally the result of pelvic surgery, radiation, or gynaecological malignancy in the developed world. In developing countries, the condition arises mainly from obstetric accidents, usually obstructed labour or instrumental vaginal delivery (Dorairajan and Hemal, 2009). Patients with VVF usually present persistent urinary leakage through the vagina. Together with medical, surgical, and obstetric background, gynaecological examination is essential to detect the fistula and assess vaginal tissue condition. Cystoscopy is crucial to determine the site of the fistula in relation to the bladder trigone and the continent mechanism, to assist with the decision making. Laparoscopic treatment of VVF started in 1994 and is currently a widely used technique (Smith et al., 2019).

The timing of the repair, as well as the definition of early and late approach, are still debated, and most data in literature suggest a delayed approach (Malik et al., 2018; Stamatakos et al., 2014). Timing for fistula repair hinges on factors such as tissue quality, extent of inflammatory/necrotic reaction, and VVF aetiology. There are large series of obstetric fistulae that report high success rates with early repairs (Waaldijk, 2004), even if a delayed approach is recommended when the quality of the tissue is compromised. Conversely, prompt intervention could be beneficial for gynaecological fistulas (Blandy et al., 1991).

We present a post-hysterectomy VVF successfully repaired laparoscopically, 30 days after the primary surgery.

## Patients and methods

We report a case of a 54-year-old patient, G7P3, with a clinical history of three extra uterine pregnancies - one of those treated by a laparoscopic right salpingectomy -, a laparoscopic tubal sterilisation and a hysteroscopic resection of endometrial polyp held in January 2023. The patient underwent a laparoscopic total hysterectomy with left salpingectomy in July 2023, due to persistent metrorrhagia. The postoperative course was uneventful. Three weeks after the surgery she presented at the emergency room with continuous urine discharge. A simple 1.5 cm vesical-vaginal fistula was diagnosed according to the clinical presentation. An early laparoscopic repair was scheduled 30 days after the primary surgery.

## Results

Under standard general anaesthesia, a cystoscopy was conducted before laparoscopy. The fistula origin was identified at the trigone, in the midline, 2 centimetres from ureteral orifices. Laparoscopy was performed in dorsal lithotomy position, with a 17-degree Trendelenburg incline and the legs supported by stirrups. Pneumoperitoneum pressure was 10mmHg. Rotating bipolar, monopolar scissors, atraumatic grasper and two needle drivers were used.

After adhesiolysis, methylene blue was injected inside the bladder, enhancing the fistula’s edges and a 4-centimetre vaginal dehiscence. Vesico- vaginal dissection was performed by pushing down the anterior vaginal wall, obtaining 4-centimetre free flaps for suturing. The whole procedure was carried out using an extravesical approach.

To revitalise the borders, the previous stitches, the fibrotic and the inflammatory tissues were excised. A dual layer of interrupted, absorbable, monofilament 3-0 cross-stitches was applied to the bladder with extracorporeal knotting.

Iodine was administered on the vaginal cuff. A single layer of interrupted, absorbable monofilament 0, was applied to the vagina, with extracorporeal knotting. At the end of the procedure, 300 cc of blue methylene was injected into the bladder, and no leakage was observed. Finally, the omentoplasty was performed. The omental flap with preserved vascularisation was placed on the vaginal cuff to separate the sutures and prevent future adhesions. The total operative time was 98 minutes. The patient was discharged on postoperative day 5 with the urinary catheter in place.

Two weeks later the catheter was removed, no post-voiding residual volume was detected. At the 4-month postoperative follow-up no complaints nor complications were reported.

## Discussion

We described a successful case of early laparoscopic approach for the treatment of an iatrogenic simple VVF. Since the incidence of this complication is rare - 0.1 to 0.2% (Zumrutbas et al., 2014) -, and the symptoms are debilitating, prompt diagnosis and appropriate management are crucial.

First of all, the previous hysterectomy and the leakage of urine represented the typical features reported in the literature for the same condition (Dorairajan and Hemal, 2009; Malik et al., 2018). Additional diagnostic exams were not necessary. However, as suggested (Núñez Bragayrac et al., 2015) , a pre-operative cystoscopy to assess fistula characteristics and its relationship to the bladder and ureteral orifices was carried out in the operative room before surgery.

Since the primary surgery was performed laparoscopically, a minimally invasive approach was adopted for the treatment of the complication. As reported, abdominal surgery is usually preferred in patients with a large (>3 cm) or supra-trigonal fistula or fistulas in proximity to ureteric orifices, especially in patients with multiple complicated or recurrent VVFs after transvaginal repair (Sotelo et al., 2012).

Moreover, the advantages of the laparoscopic approach include decreased postoperative pain, early resumption of feeding, and better aesthetic result in comparison with open surgery (Núñez Bragayrac et al., 2015) .

An early approach for repairing the VVF was already described by Blandy et al. (1991), however, there is still no consensus on the timing of surgical repair. Regarding VVFs, the categorisation according to the aetiology and tissue quality is mandatory. In obstetrical VVFs, there is often tissue necrosis and concurrent ongoing infection, rendering an immediate approach hazardous and fraught with morbidity. The same cannot be said for gynaecological fistulas, which are mostly aseptic. Another reason for early repair is that there is no oedematous adhesion and no tension during surgical separation and suturing (Wang et al., 2023). Hence, the vesico-vaginal dissection in the reported surgery, was carried out without any difficulty.

Finally, the role of the omental flap should be underlined. To date, there are no well-defined criteria for the use of flap interposition. It is a matter of pre-and intraoperative assessment of tissue quality and other factors such as nutritional status, local inflammation/infection, and risk of fistula recurrence. Flaps are mostly used in the setting of radiated tissues, large fistulas, multiple coexisting fistulas, or where there are multiple risk factors for failure of primary repair. Obstetric fistulas often benefit from flaps due to their predilection for substantially associated ischemic tissue beyond what is clinically evident on initial inspection. In order to achieve the best functional result, it is important to preserve the vascularisation of the flap. By definition, tissue flaps bring along with them a blood supply that continues to sustain them at the target site (Margules and Rovner, 2019).

Given that peculiarity, the selection of the flap site and the dissection should be thoroughly assessed, as shown in the video.

## Conclusions

Urinary tract fistulas represent a complex group of pathologies that present significant management challenges. As reported, early repair of an iatrogenic vesico-vaginal fistula is feasible and safe. Further randomised studies are needed to standarise the technique.

## Video scan (read QR)

https://vimeo.com/891874637/cb923cc7ef?share=copy

**Figure:**